# Evaluation of some heavy metals residues in batteries and deep litter rearing systems in Japanese quail meat and offal in Egypt

**DOI:** 10.14202/vetworld.2017.262-269

**Published:** 2017-02-28

**Authors:** Ali M. Ahmed, Dalia M. Hamed, Nagwa T. Elsharawy

**Affiliations:** 1 Department of Food Hygiene, Faculty of Veterinary Medicine, Suez Canal University, Egypt; 2 Department of Poultry and Rabbit Medicine, Faculty of Veterinary Medicine, Suez Canal University, Egypt; 3 Department of Food Hygiene, Faculty of Veterinary Medicine, New Valley, Assiut University, Egypt

**Keywords:** batteries, deep litter, heavy metals, offal, poultry meat

## Abstract

**Aim:**

The main objectives of this study were for comparing the effect of batteries and deep litter rearing systems of domesticated Japanese quail, *Coturnix coturnix japonica*, on the concentration levels of cadmium, copper, lead, and zinc from the quail meat and offal in Ismailia, Egypt.

**Materials and Methods:**

A total of 40 quail meat and their offal samples were randomly collected from two main quail rearing systems: Battery (Group I) and deep litter system (Group II) for determination of concentration levels of cadmium, copper, lead, and zinc. In addition, 80 water and feed samples were randomly collected from water and feeders of both systems in the Food Hygiene Laboratory, Faculty of Veterinary Medicine, Suez Canal University for heavy metals determination.

**Results:**

The mean concentration levels of cadmium, copper, lead, and zinc in Group I were 0.010, 0.027, 1.137, and 0.516 ppm and for Group II were 0.093, 0.832, 0.601, and 1.651 ppm, respectively. The mean concentration levels of cadmium, copper, lead, and zinc in quail feed in Group I were 1.114, 1.606, 5.822, and 35.11 ppm and for Group II were 3.010, 2.576, 5.852, and 23.616 ppm, respectively. The mean concentration levels of cadmium, copper, lead, and zinc in quail meat for Group I were 0.058, 5.902, 10.244, and 290 ppm and for Group II were 0.086, 6.092, 0.136, and 1.280 ppm, respectively. The mean concentration levels of cadmium, copper, lead, and zinc for liver samples in Group I were 0.15, 8.32, 1.05, and 3.41 ppm and for Group II were 0.13, 8.88, 0.95, and 4.21 ppm, respectively. The mean concentration levels of cadmium, copper, lead, and zinc in kidney samples for the Group I were 0.24, 4.21, 1.96, and 4.03 ppm and for Group II were 0.20, 5.00, 1.56, and 3.78 ppm, respectively. Kidney had the highest concentration levels of heavy metals followed by liver then muscles. The highest concentration levels of copper were observed in liver samples. The order of the levels of these trace elements obtained from the four different quail organs is Ca > Pb > Zn > Cu. Lead and cadmium concentration levels in quail meat samples were exceeded the Egyptian standardization limits and suggesting a health threat from lead and cadmium to the quail consumers.

**Conclusion:**

Battery rearing system is more hygienic than deep litter system from the point of heavy metals pollution of water and feeds of quail. Feed samples from battery system had means concentration levels of lead not significantly higher (p>0.05) than those samples from deep litter system. Meanwhile, water samples from battery system had means concentration levels of cadmium, copper, and zinc significantly higher (p>0.05) than those samples from deep litter system. Quail may carry health risks to consumers.

## Introduction

Domesticated Japanese quail (*Coturnix coturnix japonica*) is medium-sized fowl which more eaten by Egyptians due to its low cost, rapid growth, early onset of lay, high reproduction rates, and low feed intake. Japanese quail meat is high protein content (19.6%), low-fat content (12.1 mg/100 g meat), low calorific value (192 g kcal/100 g meat), the highest amount of omega 3 fatty acids and vitamin A [1].

Increasing consumption of Japanese quail in Egypt promotes quails farms which using two rearing systems; deep litter system “Six quails can be reared in a square feet of floor space, quails transferred to cages after 2 weeks which gaining high body weight,” while in battery system rearing; the space area of each unit is about one foot in width and six feet in length which subdivided into six subdivisions. Cages arranged up to six layers for saving area, each row containing about 4-5 cages. Cleaning the cage was been performed by fixation of mobile wooden plates on the bottom of the cage, feed troughs fixed in the front of cages while water troughs placed backward [2].

Heavy metals contamination is one of the major concerns worldwide, which influences the practical and structural integrity of an environment and cannot be avoid their direct and indirect effects on animal and human health. These contaminants may lift their residues in animal and birds tissues. In quail, heavy metals residues cannot be seen, smelled or tasted, although its amount in organs like kidney, liver is higher than muscle [3,4]. Bioaccumulation of heavy metals and their long biological half-lives in the quails consumers’ bodies leading to dangerous side effects which may reach to sub-lethal or even lethal impact by long-run consumption of very low concentrations above the body requirements[5-10].

According to Duruibe *et al*. [11], cadmium, copper, lead, and zinc are toxic elements and dangerous to quail even at low concentrations when ingested over an extended period [12]. Cadmium toxic effects induced the detoxification in liver and kidney enzymes, which may lead to kidney dysfunction, hepatic injury, lung damage, and hypertension reaching to teratogenic effect [13-15]. Overdoses of copper, causes liver oxidative damage, and hepatic granular degeneration [16]. Lead poisonous has neurotoxic effect, cellular inactivation, binds to gastrointestinal enzymes, and renal systems [17-19]. Low zinc intake may lead to loss of appetite, slow healing of wounds, immunity depression, retarded growth of male sex organs while; overdose of zinc may cause abdominal cramps, vomiting, and nausea [20,21].

Domesticated quail had been rearing in Ismailia city, Egypt on battery and deep litter system for meat production. Although many researchers try to evaluate the heavy metals levels in Japanese quail meat, the available data about the trace element levels in Japanese quail at Ismailia city, Egypt are still scarce [22-24]. Therefore, this study was to determine the concentration ranges of cadmium, copper, lead, and zinc in quail meat, their offal, water and feeds from Ismailia town, reared on the each system.

## Materials and Methods

### Ethical approval

The Animal Rights and Ethical Use Committee of Suez Canal and Assiut Universities have approved this study.

### Study area

A cross-sectional study was directed in Ismailia quails butchers to determine the concentration levels of heavy metals deposits in meat and consumable offal of quails.

### Samples collection

#### The first part (preliminary study)

A total of 80 water and feed samples (40 each) were randomly collected equally from quails waters and feeders of battery Group I and deep litter systems (Group II). At each rearing system, each water sample (100 ml) was collected using clean tube sampler and kept refrigerated on icebox. Furthermore, each feed sample (100 g) was collected and kept in clean polyethylene bags, and then all samples were transferred into the Food Hygiene Laboratory, Faculty of Veterinary Medicine, Suez Canal University, for heavy metals determination.

#### Second part (main study)

A total of 60 of quail, liver and kidney samples (20 each) were randomly collected from two different locations: Markets and farms of Ismailia city. All fresh quail carcasses weighted 250 g±15 included their liver and kidney were collected in polyethylene bags and kept in icebox then immediately transferred to the Food Hygiene Laboratory, Faculty of Veterinary Medicine, Suez Canal University, for heavy metals determination.

### Samples preparation

About 25 g of each sample was well homogenized into a clean silica dish. Then, 25 ml of 20% sulfuric acid (b) was added. The sample was thoroughly mixed with a glass-stirring rod ensuring that the acid wets all material. The stirring rod was rinsed with water into silica dish. The contents of the dish were thoroughly dried on an oven at 110°C. Then, the dish was transfer into a furnace set at 25°C. The temperature was slowly raised to 500°C for about 6-8 h. The dish was removed and cooled.

### Samples analysis

The digest, blank, and standard solutions were aspirated by the atomic absorption spectrophotometer [25]. Analysis was performed by Flame Atomic Absorption Spectrophotometer (VARIAN, Australia, Model AA240 FS) for determination of copper, calcium, and iron. The graphite furnace atomic absorption spectrophotometer (VARIAN, Australia, Model AA240 Z) was used for determination of lead and cadmium.

The heavy metals concentrations were calculated according to the following equation:

Element, ppm=R × D ÷ W

Where, R=Reading of the digital scale of AAS,

D=Final volume of the prepared sample,

W=Weight of the sample.

### Statistical analysis

Data collected were presented as mean, minimum concentration, maximum concentration, and standard error and were subjected to one-way analysis of variance. Differences between the means were tested by Duncan tests. The level of significance was chosen at p<0.05 and the results are presented as mean to assess whether heavy metals varied significantly between different samples. All statistical calculations were performed with SPSS 9.0 for Windows [26].

## Results

### The first part (preliminary study)

#### Heavy metals residues in water samples

The obtained results in Table-1 revealed that the mean residual concentration levels of cadmium, copper, lead, and zinc in water samples for the Group I were 0.010, 0.027, 0.137, and 0.516 ppm and for Group II were 0.093, 0.832, 0.601, and 1.651 ppm, respectively. The water samples from battery system had means concentration levels of cadmium, copper lead, and zinc significantly higher (p>0.05) than those samples from deep litter system.

**Table-1:** Statistical analytical results of mean concentration levels of cadmium, copper, lead, and zinc in water samples and feeds samples (n=20).

Groups	Water samples				Feed samples		
	
	Metals	Minimum	Maximum	Mean±SE	Minimum	Maximum	Mean±SE
Group I	Cadmium	0.001	0.014	0.010^a^±0.002	0.640	1.320	1.114^a^±0.128
	Copper	0.014	0.036	0.027^a^±0.004	0.990	2.070	1.606^a^±0.174
	Lead	0.011	0.319	0.137^a^±0.015	5.340	6.290	5.822^a^±0.196
	Zinc	0.146	0.963	0.516^a^±0.134	30.25	38.90	35.11^b^±1.564
Group II	Cadmium	0.011	0.400	0.093^b^±0.023	1.940	3.590	3.010^b^±0.291
	Copper	0.056	1.348	0.832^b^±0.240	1.680	3.190	2.576^b^±0.258
	Lead	0.090	0.508	0.601^b^±0.038	5.170	7.210	5.852^ab^±0.396
	Zinc	0.241	5.210	1.651^b^±0.915	20.36	28.36	23.62^a^±1.345

Group 1: Battery system, Group 2: Deep litter system, Mean in the column has different letter is significantly difference (p≤0.05). SE=Standard error

The World Health Organization [27] set recommended limits to drinking water for livestock; these permissible limits are for cadmium, copper, and lead are 0.05, 0.5, and 0.2, respectively. According to these limits, the concentration levels of heavy metals in Group I were set within the permissible limit for lead and were higher for cadmium and copper residues. For water samples in Group II, the concentration levels of lead, cadmium, and copper were founded higher than the permissible limits.

Zinc was 1.651 which higher scientifically in Group II in compared to Group I. Unfortunately, there is no permissible limit or recommended guide for the concentration levels of zinc in water.

#### Heavy metals in feeds

The obtained results in Table-1 revealed that the mean residual concentration levels of cadmium, copper, lead, and zinc in feed samples for the Group I reared in battery system were 1.114, 1.606, 5.822, and 35.110 ppm, respectively, and for Group II reared in deep litter system were 3.010, 2.576, 5.852 and 23.616 ppm, respectively. Feed samples from battery system had means concentration levels of lead not significantly higher (p>0.05) than those samples from deep litter system. Meanwhile, feed samples from battery system had means concentration levels of cadmium, copper, and zinc significantly higher (p>0.05) than those samples from deep litter system.

### Second part (main study) heavy metals in quail meat and their offal

#### Cadmium

The obtained results in Table-2 showed that the mean residual concentration levels cadmium in quail meat, liver, and kidney samples for the Group I samples were 0.058, 0.15, and 0.24 ppm, respectively, for Group II sample were 0.086, 0.13, and 0.20 ppm, respectively. Cadmium highly accumulated in poultry kidney then liver more than muscle [3,28-30]. Quail meat samples from markets at Ismailia city had means concentration levels of cadmium significantly higher (p>0.05) than farms samples.

**Table-2:** Statistical analytical results of mean concentration levels of cadmium and copper in quail meat samples (n=20).

Items	Cadmium						Copper					
	
	Group I			Group II			Group I			Group II		
			
	Meat	Offal		Meat	Offal		Meat	Offal		Meat	Offal	
			
		Liver	Kidney		Liver	Kidney		Liver	Kidney		Liver	Kidney
Minimum	0.010	0.10	0.12	0.010	0.09	0.16	4.300	2.40	1.89	3.580	2.55	1.14
Maximum	0.110	0.28	0.41	0.041	0.23	0.36	7.800	9.25	6.63	7.510	9.14	6.34
Mean±SE	0.058^a^±0.02	0.15^a^±0.02	0.24^b^±0.01	0.086^b^±0.01	0.13^ab^±0.01	0.20^ab^±0.01	5.902^a^±0.59	8.32^a^±1.80	4.21^c^±1.52	6.092^ab^±0.54	8.88^ab^±1.32	5.00^cd^±10.2

Group I: Battery system, Group II: Deep litter system, Mean in the column has different letter is significantly difference (p≤0.05). SE=Standard error

**Figure-1:**
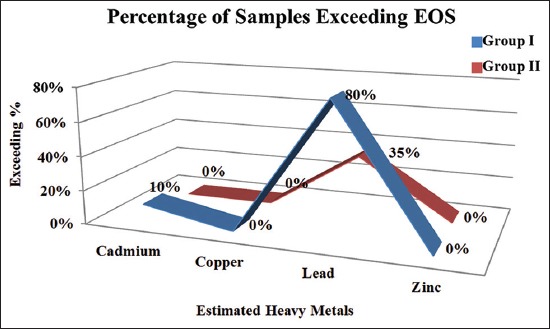
Frequency distribution of concentration levels of cadmium, copper, lead, and zinc in the quail meat with compared to Egyptian standard.

The Egyptian Organization for Standardization and Quality Control [31] sets a permissible limit for cadmium in poultry meat which must be not exceeding than 0.05 mg/kg. From results in Figure-1, 2 (10%) and 0 (0%) of the quail meat samples between Group I and Group II, respectively, were exceeded the permissible limit of the Egyptian standard meanwhile 18 (90%) and 20 (100%) of the samples, respectively, were within the permissible limit for cadmium.

#### Copper

The obtained results in Table-2 showed that the mean residual concentration levels of copper in quail meat, liver, and kidney samples for the Group I samples were 5.902, 8.32, and 4.21 ppm, respectively, while Group II samples were 6.092, 8.88, and 5.00 ppm, respectively. Copper highly accumulated in poultry liver then kidney more than muscle [32-34]. Quail meat samples from Group I at Ismailia city had means concentration levels of copper not significantly higher (p<0.05) than those samples from farms.

The Egyptian Organization for Standardization and Quality Control [31] sets a permissible limit for copper in poultry meat, which must be not exceeding than 15.00 mg/kg. From results in Figure-1, 20 (100%) of the samples, respectively, were within the permissible limit for copper.

#### Lead

The obtained results in Table-3 showed that the mean residual concentration levels lead in quail meat, offal (liver and kidney) samples for the Group I were 0.244, 1.05 and 1.96 ppm, while samples of Group II from the farms were 0.136, 0.95 and 1.56 ppm, respectively. The kidney was the target organ of the lead accumulation followed by liver and muscle [3,28-30]. Meat samples from Group II at Ismailia city had means concentration levels of lead significantly higher (p>0.05) than those samples from Group I.

**Table-3:** Statistical analytical results of mean concentration levels of lead and zinc in quail meat samples (n=20).

Items	Lead						Zinc					
	
	Group I			Group II			Group I			Group II		
			
	Meat	Offal		Meat	Offal		Meat	Offal		Meat	Offal	
			
		Liver	Kidney		Liver	Kidney		Liver	Kidney		Liver	Kidney
Minimum	0.090	0.84	0.98	0.070	0.70	0.90	1.050	1.66	1.20	1.110	2.11	1.90
Maximum	0.730	2.54	3.02	0.450	2.01	2.14	1.420	532	6.21	1.380	6.11	5.55
Mean±SE	0.244^a^±0.05	1.05^a^±0.08	1.96^a^±0.07	0.136^b^±0.04	0.95^b^±0.04	1.56^d^±0.05	1.290^a^±0.06	3.41^a^±0.25	4.03^c^±1.01	1.280^a^±0.06	4.21^ab^±1.10	3.78^cd^±0.74

Group I: Battery system, Group II: Deep litter system, Mean in the column has different letter is significantly difference (p≤0.05). SE=Standard error

The Egyptian Organization for Standardization and Quality Control [31] sets a permissible limit for lead in poultry meat, which must be not exceeding than 0.10 mg/kg. From results in Figure-1, 16 (80%) and 7 (35%) of the quail meat samples, respectively, from Group I and Group II were exceeded the permissible limit of the Egyptian standard meanwhile 4 (20%) and 13 (65%) of the samples, respectively, were within the permissible limit for lead.

#### Zinc

The obtained results in Table-3 showed that the mean residual concentration levels zinc in quail meat, liver, and kidney samples for the Group I samples were 1.290, 3.41, and 4.03 ppm, respectively, for Group II samples were 1.280, 4.21, and 3.78 ppm, respectively. Quail meat samples from Group I at Ismailia city had means concentration levels of zinc not significantly higher (p<0.05) than Group II. Zinc highly accumulated in poultry kidney then liver more than muscle [3].

Zinc is essential nutrients for animals. Zinc used as dietary ingredients Kottferova *et al*. [35]. WHO [27] sets a permissible limit for zinc in poultry meat, which must be not exceeding than 100 mg/kg. From results in Figure-1, 20 (100%) of the two groups samples were within the permissible limit for zinc.

## Discussion

The concentrations of various metals in meat are critical because these contaminants have deleterious effects on consumers. Many illnesses and diseases such as cancer and hypertension have been associated with increased concentrations of cadmium, copper, lead, and zinc in meat consumers’ organs.

### The first part (preliminary study) heavy metals residues in water and feeds samples

Water and feed samples from battery system had means concentration levels of lead not significantly higher (p>0.05) than those samples from deep litter system. Meanwhile, water and feed samples from battery system had means concentration levels of cadmium, copper, and zinc significantly higher (p>0.05) than those samples from deep litter system.

The obtained results clarify the presence of different heavy metals (cadmium, copper, lead, and zinc) in various levels as in Table-1. Nearly, Okoye *et al*. [36] who found about 0.038-0.463 cadmium, 6.52-14.20 copper, 1.10-7.85 lead, and 34.038-49.950 zinc in Nigeria poultry water and feeds recorded similar values. However, Raj *et al*. [37] found higher results in India were recorded about 0.174 mg/L Cd, 0.002 mg/L Cu, 1, 0.001 mg/L Pb, and 0.011 mg/L Zn. On the other hand, Abdou *et al*. [38] noted lower levels of 0.004-0.029 ppm Cd, 0.015-0.057 ppm Cu, 0.247-0.379 ppm Pb, and 0.008-0.164 ppm Zn in other cities in Egypt drinking water, while Suganya *et al*. [39] recorded about 0.5 ppm Cd, 300 ppm Cu, 30 ppm Pb, and 1000 ppm Zn of poultry feeds in India.

It could be interpreted that heavy metals are existed in higher level in environment consequently, which may lead to bioaccumulation in water and feeds. That might attribute to water pollution, manure, and drainage system which hazard may reflect on quail production as bird consumes from 15 to 20 time’s water as much as they consume feed [27]. The obtained variances were might attribute to contamination of water during slaughter with manures similar results were clarified by Maff [40] who found that heavy metal concentration at soil levels for plant.

### Second part (main study) heavy metals in quail meat and their offal

#### Cadmium

Tissue Cd concentrations in animals are closely related to Cd levels in feedstuffs and the duration of Cd load [41]. Nutritional and vitamin status, such as iron status, age and sex and a wide range of factors controlling absorption and accumulation of Cd in tissue [42].

The obtained results came with agree with those obtained by Herzig *et al*. [43] in Czech Republic who found 4.99±1.57, 0.558±0.60, and 0.052±0.008 mg/kg in liver, kidneys, and muscle, respectively. Mariam *et al*. [22] who recorded 0.3 mg/kg in Egyptian poultry meat recorded higher results of cadmium concentration levels in quail meat. However, Benouadah *et al*. [2] reported about 17.77-19.55 ug/kg in kidney, 5.45-11.79 ug/kg in liver, and 4.69-10.53 ug/kg in meat of Algerian poultry. In addition, the lower prevalence rates have recorded in other countries; 0.004-0.124 µg/g in Iraq [42] and 0.01-5.68 mg/kg in Nigeria [23].

Cadmium is poorly absorbed in the body, but as soon as absorbed and slowly excreted, like different metals, and accumulates within the kidney causing renal harm. The kidney of food animals is a major source of cadmium in the diet even though lower degrees are located in lots of foods; cadmium is endocrine demanding substance and may cause the development of prostate and breast most cancers in addition to kidney and skeletal damage in humans [44,45].

#### Copper

The quantity of copper ingested, interval of exposure, animal age and breed all that are factors controlling copper accumulation in the organs [46,47]. Copper is an essential trace element that is toxic at excessive doses.

Nicolas *et al*. [29] obtained meanly similar results for copper concentration levels in quail meat and their offal in Nigeria. Lower results of copper concentration levels in quail meat were recorded by Khan *et al*. [34], who noted 0.357, 1.35 ppm Cu in Pakistani poultry meat and liver, respectively, in Egypt Donia [3] recorded about 6.64±4.35, 3.32±3.68, and 2.84±3.68 mg/kg, (2.84-6.64) in quail liver, kidney, and meat, respectively. Akan *et al*. [6] also recorded 0.1-1.44 ug/g of in liver, kidney, and meat of chicken from Nigeria. Skalicka *et al*. [30] who found about 9.346 mg/kg and 4.822 mg/kg in liver and muscle of Japanese quails recorded higher values, respectively, in Poland; while in Malaysia, Abduljaleel *et al*. [48] reported about 24.77 and 8.6 mg/kg in the liver and quail meat, respectively.

Long exposure to copper leading to Wilson’s and Menke’s diseases which characterized by irritation of the eyes, nose and mouth, stomachaches, headaches, dizziness, vomiting, and diarrhea, also liver and kidney may affect. Daily recommended intake of copper is 2 mg, and as little as 10 mg of copper will have a poisonous impact [4,23,49].

#### Lead

Lead is one of the most toxic heavy metals having unknown biochemical benefits [3,4,30]. High residual levels of lead in quail meat samples obtained from either Group I or Group II caused by many sources such as industry. High metals levels in poultry products emanate mainly from contamination of feeds and water sources. Quail meat samples had high concentration levels of lead due to water used during quail processing for human consumption, which found be polluted with lead in different area at Ismailia city.

Skalická *et al*. [30] obtained meanly similar results for lead concentration levels in quail meat and their offal. Higher results of lead concentration levels in quail meat were recorded in Egypt by Donia [3], who reported about 1.63±0.18, 6.17±0.59, and 1.06±0.38 mg/kg in quail meat respectively [50], detected about 2.75 and 2.8 μg/g for liver and muscle from Qena and Assiut broilers, in addition, those observed by Khan *et al*. [34] in Pakistan who found about 1.234 and 1.797 mg/kg of Pb in liver and meat of Gallus domesticus chicken.

On the other hand, another study revealed lower results in Turkey by Uluozlu *et al*. [24] who reported 0.01-0.40 ug/g for Pb in chicken; Abduljaleel *et al*. [48] who recorded 0.55 and 0.47 mg/kg in the liver and quail muscle in Malaysia and Ghimpeteanu *et al*. [51] who noted 0.006 mg/kg Pb in poultry liver in Belgium and Romania.

The results have revealed that the lead concentration in meat is alarming and indicates the high levels of lead pollution in the environment. The major source of lead pollution is exhaust gases, which arise from agents, added in gasoline by automobile.

The poisonous consequences of lead established in studies on people exposed to lead within the course of their work. The exposure to excessive levels of lead for a short period can cause brain harm, paralysis (lead palsy), anemia and gastrointestinal signs and symptoms, while the long exposure to lead can cause damage to the immune system, reproductive, nervous systems, and kidneys. The most dangerous effect of exposure to low lead level is on intellectual improvement in young kids; lead crosses the placental barrier and accumulates in the fetus. Thus infants and young children are greater affected than adults to the lead poisonous consequences. Even short exposures of young children to low levels of lead must take into consideration due to its effect on neurobehavioral development [52-54].

#### Zinc

Zinc is an essential element, playing an important role as coenzymes in the body, also needed for the tissues repairing and growth, forming connective tissues, skin, bones, teeth, hair, and nails [3,34,55,56]. Donia [3] recorded higher results of zinc concentration levels in quail meat in Egypt in quail organs were 18.9, 25.5 and 9.82 mg/kg in liver, kidney and quail muscle respectively [30], in Slovak recorded 25.5 mg/kg and 8.11 in kidney and quail muscle. Abduljaleel *et al*. [48] in Malaysia reported in Japanese quails about 50.607 and 51.076 mg/kg in liver and muscle. On the other hand, lower results obtained in Iraq by Reem *et al*. [42] who found about 3.266 mg/kg Zn from poultry liver.

Zinc added to poultry diets to improve immunity, weight gain. Zinc toxicity leading to pneumonia mortality in children [57].

## Conclusion

The results of this work indicated the heavy metals concentrations in Domesticated Japanese quail; *C. coturnix japonica* quail were statistically significant from two main quail rearing systems: Battery (Group I) and deep litter system (Group II) for determination of concentration levels of cadmium, copper, lead, and zinc. The order of the levels of these trace elements obtained from the four different quail organs is Cd > Pb > Zn > Cu. Kidney had the highest concentration levels of heavy metals followed by liver then muscles for Cd, Pb and Zn, but the highest concentration levels of copper were observed in liver samples. Lead and cadmium concentration levels in quail meat samples were exceeded the Egyptian standardization limits and suggesting a health threat from lead and cadmium to the quail consumers. Battery rearing system is more hygienic than deep litter system from the point of heavy metals pollution of water and feeds of quail. Feed samples from battery system had means concentration levels of lead not significantly higher than those samples from deep litter system. Meanwhile, water samples from battery system had means concentration levels of cadmium, copper, and zinc significantly higher than those samples from deep litter system. We recommend the battery rearing system in quail farms and decrease the heavy metals pollution of water and feeds of quail.

## Authors’ Contributions

AMA and DMH designed the experiments, study and performed the experiments; NTE interpreted the data and wrote the manuscript. All authors read and approved the final manuscript.
